# Sudden-Onset Severe Back Pain Caused by Acute Gastric Anisakiasis

**DOI:** 10.7759/cureus.52124

**Published:** 2024-01-11

**Authors:** Risa Yamamoto, Taiju Miyagami, Yuji Nishizaki, Hinata Nishimura, Yoshiki Tsumura, Toshio Naito

**Affiliations:** 1 General Medicine, Juntendo University, Tokyo, JPN

**Keywords:** upper endoscopy, medical history taking, diagnostic excellence, back pain, anisakiasis

## Abstract

Anisakiasis is a parasitic disease that usually causes acute abdominal pain, nausea, and vomiting after the ingestion of raw seafood. We present a case of anisakiasis in an 80-year-old man who complained of sudden-onset severe back pain that was reminiscent of aortic dissection. This case shows that anisakiasis should be considered as a possible differential diagnosis in patients with not only abdominal pain but also back pain. In addition, for “diagnostic excellence,” it is essential to return to a comprehensive medical history that allows the reassessment of the diagnosis even when it differs from the initial differential diagnosis.

## Introduction

Anisakiasis is a parasitic disease caused by the nematode *Anisakis simplex*, which can generally invade the gastrointestinal wall of humans and cause strong allergic reactions [1]. The main symptoms include mild-to-severe abdominal pain not confined to specific areas, nausea, and vomiting within a few minutes to four hours after consuming raw or undercooked seafood, particularly bonito and mackerel [1]. Diagnosis and treatment are usually by endoscopy and extraction and identification of the larvae [2]. Anisakiasis has been reported in significantly higher numbers particularly in Japan, Spain, and South Korea. In recent years, the number of anisakiasis reports has been increasing in many more countries across the world [3]. In this report, we describe a case of gastric anisakiasis that presented with back pain and had a rare clinical course.

## Case presentation

An 80-year-old man with hypertension and dyslipidemia presented with severe middle back pain (Th4-8 midline of the trunk). While he was asleep, he suddenly experienced severe and dull back pain, with a numerical rating scale score of nine over his entire back area. He could not sleep well due to a feeling of dyspnea caused by persistent pain. The following day, when he presented to our hospital, he was still experiencing back pain in the entire area. His medical history included hypertension and dyslipidemia. He also reported heavy alcohol consumption habits. He did not have any other symptoms such as diarrhea or black stool, except for back pain and nausea. His only medication was angiotensin II receptor blocker (ARB) for hypertension. In addition, he had eaten raw squid sashimi prepared by a cook approximately 12 hours before the symptom onset.

At presentation, his consciousness was clear, and his other vitals were as follows: temperature, 36.5°C; heart rate, 56 beats/min; blood pressure, 148/64 mmHg (no difference between right and left); respiratory rate, 12 breaths/min; and SpO_2_, 98% at room air. The abdomen was soft and flat with no tenderness. Physical examination did not reveal a pulse deficit, aortic bruit, unequal blood pressure in either arm, or costovertebral angle tenderness. Based on the patient’s clinical history, acute aortic dissection and acute pancreatitis were suspected. In addition, acute coronary syndrome, esophageal rupture, and pulmonary embolism were considered as the differential diagnoses. Because of the history of raw fish consumption, anisakiasis was also listed as a differential diagnosis, but we considered anisakiasis less likely because it is atypical for the primary symptom to be back pain rather than abdominal pain.

**Figure 1:**
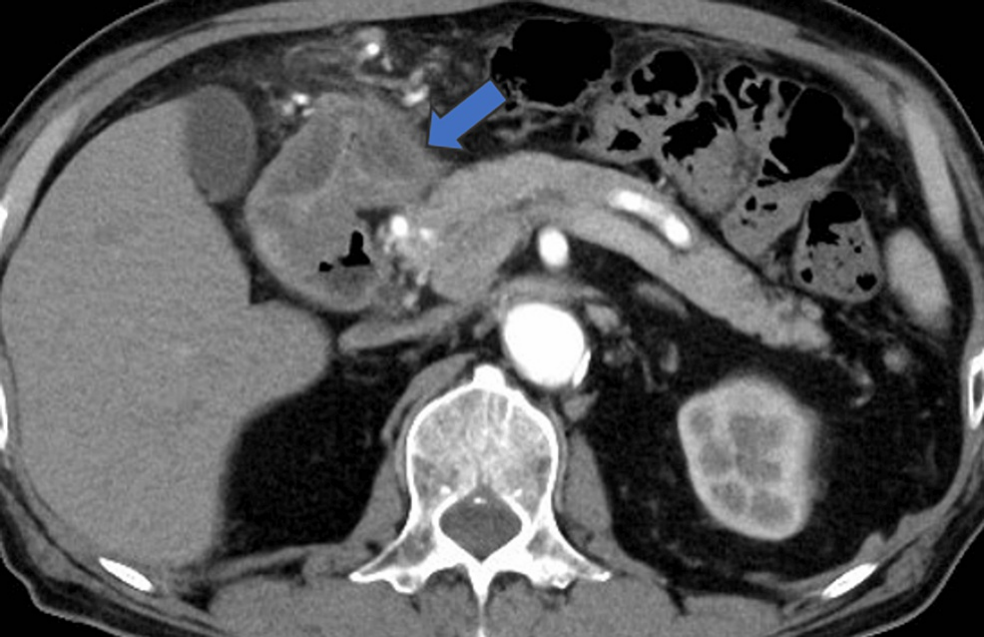
Contrast-enhanced CT Contrast-enhanced CT of the transverse section. Focal edematous wall thickening was seen in the antral measurement of the gastric body (arrow). CT, computed tomography

**Figure 2:**
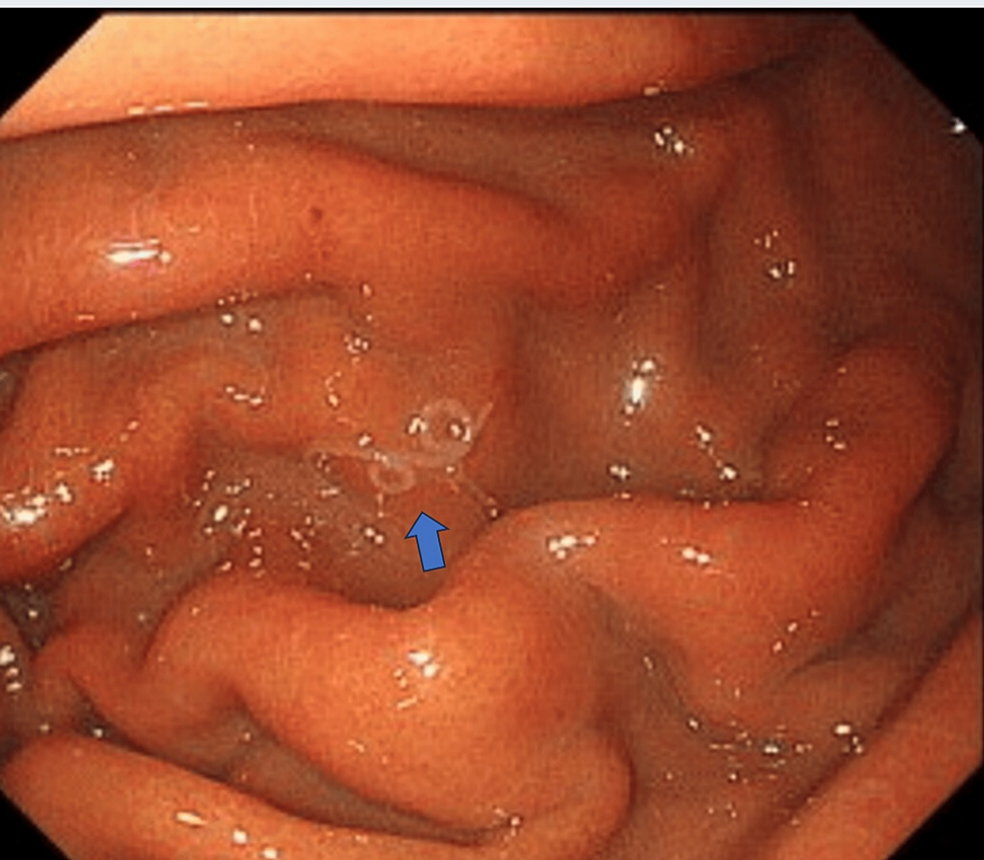
Upper endoscopy Upper endoscopy revealed Anisakis nematodes in the gastric body (arrow).

**Figure 3:**
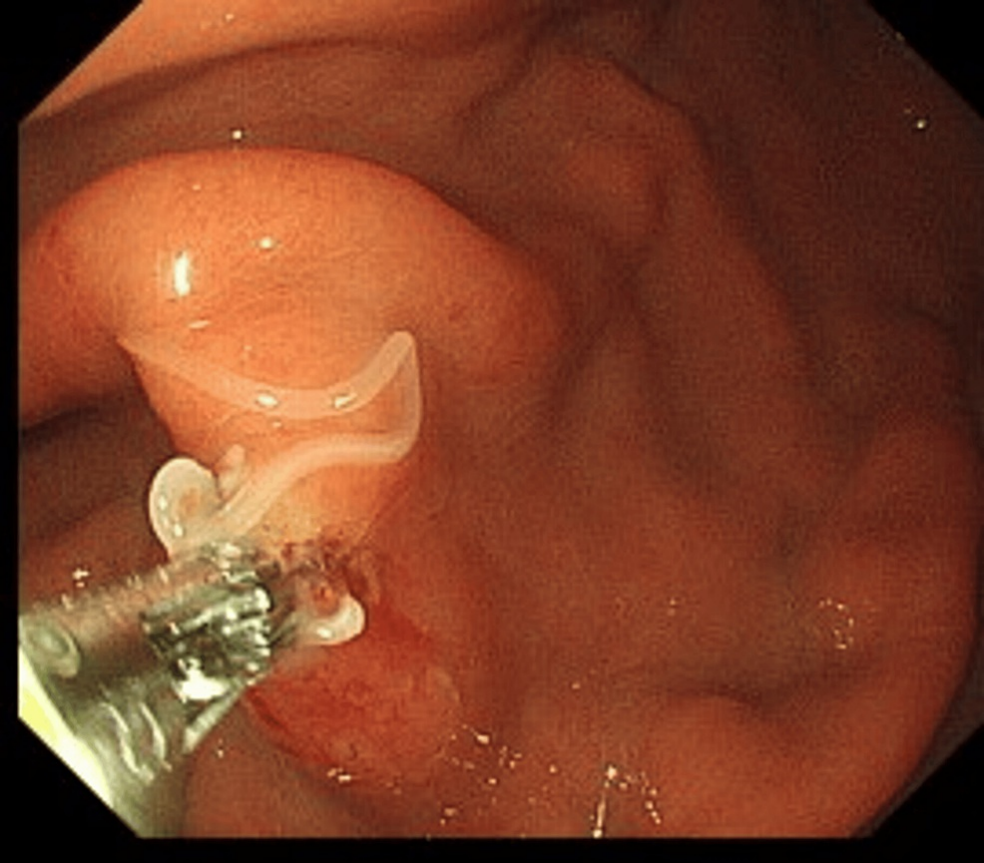
Endoscopic Anisakiasis removal Anisakis larvae were removed endoscopically with forceps.

Laboratory tests showed leukocyte levels of 5400/dL (reference range: 3900-9700) with 3.3% eosinophils, C-reactive protein level of 1.56 mg/dL (reference range: 0.0-0.29), D-dimer of 1.8 µg/mL (reference range: 0.0-1.0), serum amylase of 75 U/L (reference range: 43-124), and serum pancreatic lipase of 45 U/L (reference range: 14-56). The electrocardiogram results were also within the normal range. Contrast-enhanced computed tomography (CT) showed no findings that were suggestive of aortic dissection or pancreatitis; however, local thickening of the anterior portion of the gastric wall was observed (Figure 1). We suspected anisakiasis or gastric ulcer and performed an upper gastroscopy, which confirmed the presence of a single live *anisakid* nematode larva penetrating the gastric mucosa in the greater curvature of the lower body (Figure 2). The patient was then diagnosed with gastric anisakiasis. Endoscopically, Anisakis larvae were removed (Figure 3). There were no specific endoscopic findings other than gastric anisakiasis. Symptoms resolved immediately after endoscopic removal of the larvae.

## Discussion

We experienced an atypical gastric anisakiasis with sudden-onset severe back pain that was reminiscent of aortic dissection. Anisakiasis may sometimes require differential diagnosis from cardiovascular disorders because it has been reported to cause severe chest pain [4,5]. To the best of our knowledge, however, this is the first report of gastric anisakiasis with sudden-onset severe back pain. Other diseases were ruled out, and the symptoms disappeared after the *anisakid* nematode larvae were eradicated, suggesting that the back pain was caused by gastric anisakiasis. In addition, although the symptoms of gastric anisakiasis in this case appeared long after raw fish ingestion, few previous reports have suggested the occurrence of the disease as early as 72 hours after ingestion of raw fish [2]. Other differentiation of the pain was done retrospectively. If back pain is caused by visceral pain, urinary tract stones and cholecystitis are the most likely differentials. Both diseases could be ruled out using CT. Somatic pain was less likely because it is not aggravated by body movement. Neuropathic pain was not consistent with the location, and psychogenic pain was not consistent with the improvement of symptoms. The actual mechanism by which the gastric lesion caused back pain, and not abdominal pain, in this case, is unknown. In fact, the mechanism by which gastric anisakiasis pain develops remains largely unknown [6]. However, as with back pain caused by duodenal ulcers, it is likely that the back pain was caused by visceral pain in the stomach, which resulted in the afferent stimulation of spinal nerves [7,8]. The symptoms of the disease were likely to be associated with pain from the stomach, especially since the range of stomach-associated pain is Th5-10 and the patient had pain at Th4-8. In addition, previous studies suggested age-related changes in pain processing, such as elevation in the pain threshold, with alterations in peripheral neural elements [9]. These changes may possibly contribute to atypical pain related to anisakiasis in this case.

## Conclusions

In this case, a comprehensive interview, including a thorough dietary history, was conducted at an early stage. The possibility of gastric anisakiasis was promptly considered after excluding aortic dissection on CT imaging. For “diagnostic excellence,” it is crucial to collect the patient’s basic information as well as re-evaluate the diagnosis if it differs from the expected one. In addition, this case report highlights the need for doctors to include gastric anisakiasis in the differential diagnosis of not only abdominal pain but also back pain.
